# Case Report: Be Aware of “New” Features of Niemann–Pick Disease: Insights From Two Pediatric Cases

**DOI:** 10.3389/fgene.2022.845246

**Published:** 2022-03-11

**Authors:** Fan Chen, Shan Guo, Xuesong Li, Shengxuan Liu, Li Wang, Victor Wei Zhang, Hui Xu, Zhihua Huang, Yanqin Ying, Sainan Shu

**Affiliations:** ^1^ Department of Pediatrics, Tongji Hospital, Tongji Medical College, Huazhong University of Science and Technology, Wuhan, China; ^2^ Department of Gastroenterology, Wuhan Children’s Hospital of Tongji Medical College, Huazhong University of Science and Technology, Wuhan, China; ^3^ AmCare Genomics Lab, Guangzhou, China; ^4^ Department of Pathology, School of Basic Medicine, Tongji Medical College, Huazhong University of Science and Technology, Wuhan, China

**Keywords:** niemann-pick disease, lysosomal storage diseases, NPC1, immune, child

## Abstract

Niemann–Pick disease is a relatively common lysosomal storage disease. Cholestatic liver disease is a typical clinical phenotype of Niemann–Pick disease in infancy. The diagnosis is traditionally based on Niemann–Pick cells in bone marrow smears or liver biopsies. Treatment for cholestatic liver disease mainly includes ursodeoxycholic acid and liver protection drugs. Here, we reported two cases of Niemann–Pick disease type C, diagnosed by genetic analysis during early infancy. Besides cholestatic jaundice, the two patients also exhibited signs of immune system hyperactivity, such as elevated immunoglobulins or multiple autoantibodies, which might require the application of glucocorticoids. In addition, three novel missense variants of the NPC1 gene were identified. The findings suggest that immune activation should be considered as a “new” clinical phenotype of lysosomal storage diseases.

## Introduction

Lysosomal storage diseases (LSDs) comprise a group of more than 70 distinct genetic diseases. LSDs are characterized by the accumulation of undigested macromolecules in lysosomes of various body cells. Although these disorders are rare, they affect 1 in 5,000 live births, accounting for 14% of all inherited metabolic diseases. LSDs have similar clinical features despite different pathogenetic mechanisms (Rigante et al., 2017; Platt et al., 2018). Niemann–Pick disease is a relatively common autosomal recessive LSD. Clinical manifestations and pathogenic genes are mainly classified into types A, B, and C (Vanier, 2013). Niemann–Pick disease type C (NPC) is a progressive and fatal disorder caused by mutations in the NPC1 (OMIM 257220) or NPC2 (OMIM 607625) gene, which results in the intracellular accumulation of unesterified cholesterol. As cholesterol accumulates in cells, it affects the brain, liver, spleen, and lungs, leading to premature death. The estimated incidence of NPC is 1:100,000, with variable age of onset and clinical features (Vanier, 2010; Patterson et al., 2012; Geberhiwot et al., 2018). Perinatal manifestations of NPC often include splenomegaly, hepatomegaly, fetal ascites, or nonimmune fetal hydrops (Spiegel et al., 2009; Surmeli-Onay et al., 2013). Severe hepatic diseases, associated or not with pulmonary disease, are neonatal manifestations of NPC (Bjurulf et al., 2008; Griese et al., 2010). In comparison with the adult period, cholestatic liver disease is a typical clinical phenotype in infants. However, some affected infants may also present hypotonia and developmental delay with little hepatic and pulmonary involvement (Vanier et al., 1988). Later in childhood and adulthood, patients can present a progressive neurodegenerative disorder characterized by developmental delay, clumsiness, cataplexy, supranuclear gaze palsy, ataxia, dystonia, and progressive dementia (Vanier, 2010).

Autoimmune diseases are systemic diseases that affect approximately 5% of the population in western countries. They are characterized by chronic, systemic excessive immune activation and inflammation (Davidson and Diamond, 2001; Xiao et al., 2021). Many studies have reported an association between autoimmune phenomena and LSDs (Rigante et al., 2017). Recently, autoantibodies in NPC have been reported in several studies (Dimitriou et al., 2019; Chu et al., 2021). Herein, we reported two clinical cases of NPC in the form of cholestatic jaundice combined with immune activation in early infancy diagnosed by genetic analysis.

## Case Presentation

### Clinical Features

Two independent patients were recruited into this study after the parents signed the informed consent. Both patients presented early-onset jaundice, abnormal liver function, and hepatosplenomegaly, suggestive of cholestatic liver disease. In addition, both patients showed signs of the immune system hyperactivity, such as elevated levels of immunoglobulins.

### Patient 1

A female patient (Patient 1) was born at term after an uneventful pregnancy by cesarean delivery, with a birth weight of 3,400 g (50–75th centile). The patient was breastfed and was the first child of a nonconsanguineous Chinese couple. The patient’s mother had once miscarried before because of a bad pregnancy outcome. The father is healthy without any known comorbidity. The patient had developed progressive jaundice since the third day after birth. At 1.8 months, the patient was initially admitted to a local hospital, and hepatosplenomegaly was identified. After more than 1 month of conservative medical treatment, the patient developed yellowing of the skin and sclera, which slowly improved, while hepatosplenomegaly progressively worsened. Therefore, the patient was referred to Tongji Hospital at the age of 3.1 months. Malnutrition was documented based on a low body weight (4,400 g, < third centile). Physical examination revealed slight yellowing of the skin, increased respiratory rate, and moist rales of the lungs. The patient’s liver was 3.5 cm below the right costal margin, while the spleen was 6.0 cm below the left costal margin.

Laboratory tests showed an elevation in the serum levels of alanine aminotransferase (ALT), aspartate aminotransferase (AST), total bilirubin (TBIL), direct bilirubin (DBIL), indirect bilirubin (IBIL), *γ*-glutamyl transferase (γ-GT), total bile acid (TBA), globulin (GLB), and alpha-fetoprotein (AFP). Blood routine test results, prothrombin time (PT), activated partial thromboplastin time (APTT), and international normalized ratio (INR) were normal. Blood amino acid spectrum analysis performed by tandem mass spectrometry (MS-MS) and urinary gas chromatography-mass spectrometry (GC-MS) analysis was unremarkable. Infectious causes were also investigated. Serological markers of hepatotropic viruses, rubella virus, herpes simplex virus, Epstein–Barr virus, human immunodeficiency virus, syphilis, and toxoplasma were negative, but markers for cytomegalovirus and human parvovirus B19 were positive. Notably, immunological evaluation revealed an increased serum level of immunoglobulins tested, including IgA, IgG, and IgM. Antinuclear antibody was positive in the range of 1:3,200; moreover, anti-scl-70 antibody and IgM anticardiolipin antibody were also positive (Table 1).

**TABLE 1:** Results of blood examinations.

Serum biochemistry (References range)	Patient 1									Patient 2
	1.8 m	3.1 m	3.6 m	4.6 m^a^	6.2 m^b^	9.0 m^b^	12.4 m^b^	25.7 m^b^	32.0 m^b^	3.5 m
White blood cell count (3.50–9.50 × 10^9^/L)	5.89	7.54	9.14	NA	5.28	NA	NA	6.25	7.32	7.65
Hemoglobin (115–150 g/L)	117	117	106	NA	120	NA	NA	129	123	75
Platelet count (125–350 × 10^9^/L)	225	254	279	NA	161	NA	NA	237	283	94
Alanine aminotransferase (0–40 U/L)	134	79	58	53	40	25	27	17	17	34
Aspartate aminotransferase (0–40 U/L)	382	234	113	66	41	46	43	38	35	199
Albumin (38–54 g/L)	36.2	47.7	40.1	37.9	41.3	47.4	45.8	46.5	46.7	31.6
Globulin (20–30 g/L)	NA	35.5	35.1	19.8	18.1	17.7	21.6	18.8	23.2	17.2
Total bilirubin (3.4–17.1 μmol/L)	210.3	58.3	26.6	7.0	4.5	3.7	5.1	6.0	4.9	256.1
Direct bilirubin (0–5.0 μmol/L)	151.6	18.8	22.1	5.3	2.5	1.6	1.8	2.2	2.3	117.6
Indirect bilirubin (0–13.3 μmol/L)	58.7	39.5	4.5	1.7	2.0	2.1	3.3	3.8	2.6	138.5
Alkaline phosphatase (1–281 U/L)	409	209	245	253	200	268	283	288	240	939
γ-Glutamyl transferase (6–42 U/L)	420	504	677	251	105	37	24	12	16	64
Total bile acid (0–10 μmol/L)	130.4	132.1	68.1	14.6	10.8	4.5	4.7	4.5	5.0	142.8
Total cholesterol (<5.18 mmol/L)	NA	4.38	4.46	2.86	NA	3.66	3.81	3.47	3.34	3.20
Triglycerides (0.05–1.70 mmol/L)	NA	2.87	NA	NA	NA	NA	NA	NA	NA	1.09
Blood glucose (4.11–6.05 mmol/L)	NA	2.33	4.34	NA	NA	NA	NA	6.00	5.60	6.83
Lactate (0.50–2.20 mmol/L)	NA	2.01	1.28	NA	NA	NA	NA	NA	NA	3.70
Ammonia (11–51 μmol/L)	NA	75	80	NA	NA	NA	NA	NA	NA	39
Pyruvate (20–100 μmol/L)	NA	223.4	73.1	NA	NA	NA	NA	NA	NA	NA
Alpha-fetoprotein (≤7.0 ng/ml)	NA	50,792	NA	814.7	146.9	62.55	48.31	5.91	3.92	128,575
Immunoglobulin A (≤0.34 g/L)	NA	0.54	NA	0.39	0.40	0.25	0.26	0.30	0.45	1.45
Immunoglobulin G (2.0–6.9 g/L)	NA	15.8	NA	7.9	5.0	4.6	5.3	6.3	6.3	10.1
Immunoglobulin M (0.06–0.66 g/L)	NA	8.98	NA	1.42	0.87	1.20	1.12	1.59	2.19	2.84
Antinuclear antibody (negative)	NA	1:3,200	NA	1:1,000	1:320	negative	negative	negative	negative	NA
Anti-scl-70 antibody (0–1.0 IU/ml)	NA	4.3	NA	0.3	<0.2	<0.2	<0.2	<0.2	<0.2	NA
IgM anticardiolipin antibody (0–20.0 CU)	NA	164.6	NA	4.8	NA	NA	NA	NA	NA	NA

m, months.

a The reference range for immunoglobulin A is 0.05–0.57 g/L, for immunoglobulin G is 2.0–6.9 g/L, and for immunoglobulin M is 0.17–1.00 g/L.

b The reference range for immunoglobulin A is 0.11–1.45 g/L, for immunoglobulin G is 3.3–12.3 g/L, and for immunoglobulin M is 0.33–1.75 g/L.

NA, not available.

Abdominal ultrasound revealed hepatosplenomegaly and uneven distribution of echogenic dots, and multiple hyperechoic and hypoechoic masses within the liver parenchyma. The maximum size of the masses was 20 × 12 mm. Subsequent liver magnetic resonance imaging (MRI) showed multiple abnormal signal foci of the liver, suggesting possible fatty infiltration within the liver mass. In addition, echocardiography indicated patent ductus arteriosus, while high-resolution chest computed tomography (CT) showed severe pulmonary infection. A liver biopsy under ultrasonic guidance was performed at the age of 3.3 months. Light microscopy examination of the collected liver tissue showed some vacuolated hepatocytes with positive periodic-acid-Schiff (PAS) staining. In contrast, immunohistochemistry examination indicated that a diagnosis of hepatoblastoma could not be completely excluded because of the limited size of the tissue sample. Electron microscopy revealed intrahepatic cholestasis, giant hepatocytes, and extramedullary hematopoiesis. The collected hepatocytes had a reduced rough endoplasmic reticulum, a fuzzy mitochondrial structure, and lipid deposits. Niemann–Pick cells were not found in the liver. The bone marrow biopsy found no abnormal cells.

The patient was treated with supportive medical treatment, including liver protection drugs, ursodeoxycholic acid (UDCA), supplementary fat-soluble vitamins, and antibiotics. After ruling out contraindications and obtaining informed parental consent, methylprednisolone was started at a low dose (1 mg/kg per day) since the patient had presented the activation of the immune system and severe pulmonary infection at the age of 3.7 months. Subsequently, the patient’s clinical symptoms gradually improved. At 4 months, the patient was clinically stable and discharged. Biochemical parameters gradually improved and returned to the standard value (Table 1). At the age of 6.2 months, serum immune globulin also normalized. At the age of 9 months, the serum level of antinuclear antibodies was within a normal range. After the discontinuation of methylprednisolone at 12.4 months, there were no further abnormalities in these indicators. During the 29-month follow-up period, the patient never suffered from jaundice or pneumonia. There were no abnormalities in head MRIs or the Child Development Scale Assessment. The patient was last evaluated at 32 months. The patient’s height was 91.5 cm (25–50th centile) and weight was 14.8 kg (50–75th centile). Her liver was 2.0 cm below the xiphoid process but not below the right costal margin, while the spleen was 5.0 cm below the left costal margin. The patient is still under clinical follow-up.

### Patient 2

The second patient was also a female and was delivered at term after a protected pregnancy. She was born with a low birth weight of 1,960 g and was breastfed. The patient was the first child of nonconsanguineous and healthy parents of Chinese origin. The father’s sister died of acute jaundice hepatitis at the age of 9 years. The patient had developed progressive jaundice since the first day after birth. At the age of 3.2 months, the patient began to develop epistaxis and cough. However, there was no improvement in her clinical symptoms after treatment with drugs for 1 week. At the age of 3.5 months, the patient was admitted to Wuhan Children’s Hospital. On physical examination, severe yellowing of the skin and sclera was noticed. In addition, edema in both lower extremities and a few scattered needle-like bleeding spots were observed. The liver was 4.0 cm below the right costal margin, while the spleen was 4.0 cm below the left costal margin.

A biochemical exam revealed elevated AST, TBIL, DBIL, IBIL, alkaline phosphatase (ALP), TBA, and AFP levels. Blood routine test results showed hemoglobin level of 75 g/L (normal range: 115–150 g/L) and platelet count of 94 × 10^9^/L (normal range: 125–350 × 10^9^/L). The clotting tests revealed PT of 15.9 s (normal range: 10.2–13.4 s), APTT of 63.9 s (normal range: 25.7–39.0 s), and INR of 1.34 (normal range: 0.88–1.16). Tests for infectious causes, including hepatotropic viruses, Epstein–Barr virus, human immunodeficiency virus, and syphilis were all negative, while the test for cytomegalovirus was positive. Serum levels of the tested immunoglobulins were elevated (Table 1). At that time, autoantibodies were not tested because the doctor did not recognize the phenomenon of immune hyperactivity.

Abdominal ultrasound showed hepatomegaly 4.1 cm below the right costal margin, splenomegaly 4.7 cm below the left costal margin, and bilateral inguinal hernia. Echogenic dots were unevenly distributed. There were multiple hyperechoic masses within the liver parenchyma, and the size of the maximum mass was 6 × 6 mm. Furthermore, no bile duct dysplasia was observed. The chest radiography revealed pulmonary infection. The head CT showed no significant abnormality.

Patient 2 received antibiotics, liver protection drugs, UDCA, and supplementary fat-soluble vitamins. However, jaundice was progressive, and there was no improvement in epistaxis, cough, or edema. After 3 days of medication, her parents gave up and refused further examination and treatment. The patient was referred to Tongji Hospital at the age of 3.9 months. Physical examination showed that her previous symptoms were worsening, and she developed anuria and lethargy. Regretfully, this patient was taken home by her parents after just a genetic test, and the parents refused any other examination and treatment, including bone marrow smears or liver biopsies. A short time later, the patient died at home.

### Genetic Analysis

**FIGURE 1:**
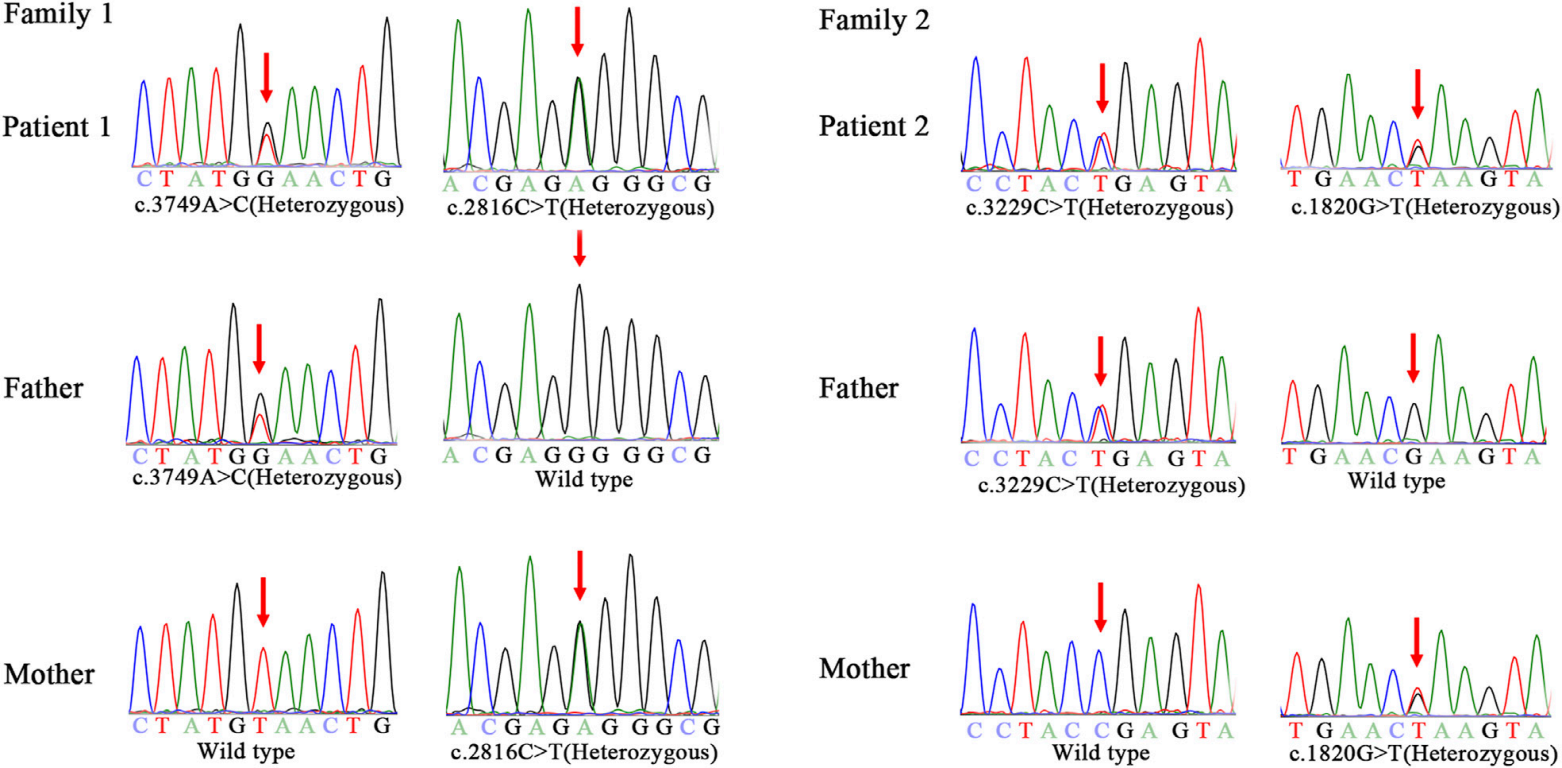
Genotyping results on NPC1 by Sanger Sequencing analysis in the two families.

**FIGURE 2:**
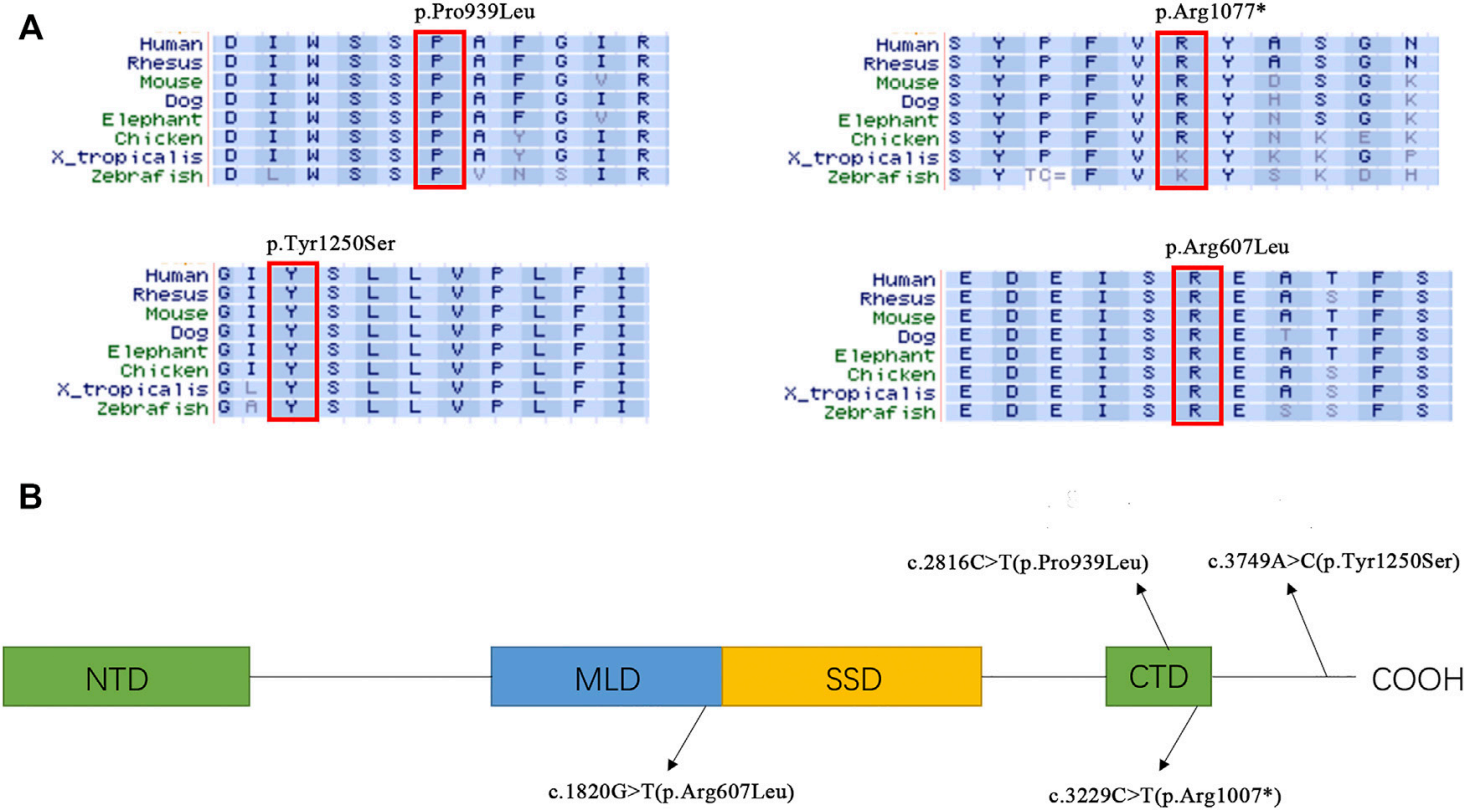
**(A)** Alignment of amino acid sequences in different organism. **(B)** Distribution of the variants in different NPC1 domains NTD, N-terminal domains. MLD, Middle Lumenal Domain; SSD, Sterol sensing domain; CTD, C-terminal Domain.

Genomic DNA of the patients and their parents was extracted from peripheral blood samples. Library preparation, custom-designed Medical Exome capture (MES, AmCare Genomic Lab), next-generation sequencing (PE 150Illumina, Inc), data alignment (Human GRCh37/hg19 assembly), and family genetic analyses were performed by in-house pipelines, with details described in previous studies (Wang et al., 2019; Li et al., 2020). Genetic diagnosis was confirmed for these two patients suspected of having NPC, and three novel missense variants (c.2816C > T, c.3749A > C, c.1820G > T) of the NPC1 gene were identified. Patient 1 carries compound heterozygous missense variant of c.2816C > T (p.Pro939Leu) inherited from the mother and c.3749A > C (p.Tyr1250Ser) inherited from the father. In the case of patient 2, the maternal missense variant is c.1820G > T (p.Arg607Leu), and the paternal variant c.3229C > T (p.Arg1077*) is a nonsense variant (Figure 1). Based on the guidelines of the American College of Medical Genetics and Genomics (ACMG) for SNV interpretation (Richards et al., 2015), these three missense variants were classified as “uncertain significance” and the nonsense variant as “likely pathogenic” (Table 2). The alignment of amino acid sequences in different organisms highly indicates the conservation of these mutations (Figure 2A). The three missense variants of these two patients have not been reported in any of the previously published cases. They were predicted to be deleterious by multiple bioinformatics tools. The nonsense variant c.3229C > T (p.Arg1077*) can lead to stop-gain at exon 21 and is predicted to cause nonsense-mediated mRNA decay (NMD) (Figure 2B). This variant has been previously reported in two individual NPC1 cases (Liu et al., 2017).

**TABLE 2:** Summary of NPC1 variants reported in this study.

Patient	cDNA	Protein	Chromosome position	Domain	Inheritance	gnomAD	Polyphen-2_HDIV Polyphen-2_HVAR	SIFT	PROVEAN	GERP++	Revel	ACMG
1	c.2816C > T	p.Pro939Leu	18:21,119,414	CTD	maternal	-	Possibly damaging Benign	D	D	Conserved	D	VUS (PM2, PP3)
	c.3749A > C	p.Tyr1250Ser	18:21,113,324	C-terminal	paternal	-	Probably damaging	D	D	Conserved	D	VUS (PM2, PP3)
2	c.3229C > T	p.Arg1077*	18:21,116,653	CTD	paternal	3.98*10^–6^	-	-	-	Conserved	-	LP (PVS1, PM2)
	c.1820G > T	p.Arg607Leu	18:21,125,051	MLD	maternal	-	Probably damaging	D	D	Conserved	D	VUS (PM2, PM3, PP3)

NM_000,271.5 for cDNA, and NP_000,262.2 for protein sequence. CTD, C-terminal *d*omain; MLD, Middle *l*uminal Domain; D, Damaging; VUS, variant of uncertain significance; LP, Likely pathogenic

## Discussion

Considering the wide range in age of onset and clinical presentations of NPC, it is a prolonged and complicated process to establish the diagnosis. Many physicians lack knowledge about the disease, so it is often missed or misdiagnosed. The presence of Niemann–Pick cells (foam cells) in bone marrow smears, spleen, or liver biopsies is one of the histological features of Niemann–Pick disease; however, these cells have not always been found (Rodrigues et al., 2006). In addition, Filipin staining in cultured skin fibroblasts is considered a key diagnostic test for NPC (Spiegel et al., 2009; Patterson et al., 2012; Mengel et al., 2013). At present, the combination of cell biology (cholestane-3β, 5α, 6β-triol, lyso-sphingomyelin isoforms, and bile acid metabolites) laboratory testing and genetic analyses is considered the first-line laboratory testing when the NPC is clinically suspected (Vanier et al., 2016; Patterson et al., 2017; Geberhiwot et al., 2018).

In this study, the two patients presented cholestatic liver disease, a typical clinical phenotype in infantile-onset Niemann–Pick disease. After excluding viral hepatitis, biliary atresia, and autoimmune hepatitis, inherited metabolic liver disease was highly suspected. However, traditional diagnostic methods failed to provide helpful information. Although liver and bone marrow biopsies of patient 1 were obtained, no significant changes were found. At that time, liver tumors could not be ruled out because of multiple liver masses and extremely high AFP levels. Therefore, next-generation DNA sequencing technologies were performed. The genetic analysis confirmed the diagnosis of NPC.

The NPC1 gene encodes NPC intracellular cholesterol transporter 1 (or Niemann–Pick C1 protein), a transmembrane protein located in the lysosome membrane. This protein interacts with NPC2 for the export of cholesterol from lysosome. Dysfunction of NPC proteins leads to the abnormal accumulation of cholesterol in lysosomes of different human tissue cells and causes Niemann–Pick syndrome with variable severity. Many studies have revealed the structure of NPC1 protein as well as the critical role of its conservative domains. The sterol-sensing domain (SSD) is the transmembrane structure of NPC1 and is the major domain for the exporting of cholesterol. The N-terminal domain (NTD) is a potential sterol-binding site for cholesterol transferred by NPC2. The middle luminal domain (MLD) is also involved in the binding process. C-terminal domain (CTD) interacts with NTD to keep it in the proper orientation for receiving cholesterol during the export process (Cologna and Rosenhouse-Dantsker, 2019; Elghobashi-Meinhardt, 2020). Three novel variants reported in this study are located in highly conserved regions related to CTD and MLD, indicating their potential roles in maintaining the NPC1 protein function. Although the p.Pro939Leu carried by Patient 1 has not previously been reported, there are several other known pathogenic variants nearby (p.Ser940Leu, p.Asp948Asn, p.Val950Met), which indicates the importance of this region (Park et al., 2003; Skorpen et al., 2012; Abela et al., 2014; Reunert et al., 2016; Pipalia et al., 2017). The variant p.Tyr1250Ser is located downstream of CTD. There are also quite a few variants reported in this C-terminal region, such as p.Val1212Leu, p.Leu1213Phe, and p.Ser1249Gly (Yamamoto et al., 1999; Garver et al., 2010; Xiong et al., 2012), indicating that there may be a specific function of the C-terminal region. However, a further functional study is necessary to better understand the mechanism by which these variants affect the NPC1 function and/or structure.

Lysosome, a subcellular organelle responsible for digestion and recycling of different macromolecules, is necessary for many cellular processes (Ballabio and Bonifacino, 2020). Therefore, it is not surprising that LSDs can display different impairments in immune responses, such as inflammation, disrupted autophagy, and autoimmunity (Rigante et al., 2017). Nevertheless, it is uncertain whether an altered immune response directly contributes to pathogenesis in LSDs. LSDs can largely be divided into two categories, where some diseases are predisposed to immunosuppression, while others are prone to immune system hyperactivity (Castaneda et al., 2008). Gaucher disease (GD), the most common LSD, is associated with immunoglobulin abnormalities (Nguyen et al., 2020). Shoenfeld *et al.* analyzed the sera of 43 patients with GD to study the presence of autoantibodies against 14 autoantigens. The immunization of naive mice with a pool of purified anti-DNA antibodies from GD patients did not result in the induction of any experimental manifestation of systemic lupus erythematosus, suggesting that these autoantibodies were nonpathogenic (Shoenfeld et al., 1995). Also, a high incidence of autoantibodies or autoimmune disorders has been reported in patients with Fabry disease (Faggiano et al., 2006; Martinez et al., 2007; Katsumata et al., 2011). The phenomenon of neuroimmune responses in Niemann–Pick disease has been widely reported, concerning mainly inappropriate microglia activation (Ledesma et al., 2011; Pressey et al., 2012; Platt et al., 2016). Sideris *et al.* showed that Niemann–Pick disease type B could coexist with autoimmune pulmonary alveolar proteinosis in an 8-year-old girl (Sideris and Josephson, 2016). Recent studies have reported the rare association of Niemann–Pick disease type B and systemic lupus erythematosus in adult cases (Murgia et al., 2015; Baya et al., 2020). Furthermore, NPC has also been linked to inflammatory bowel disease (Jolliffe and Sarkany, 1983; Steven and Driver, 2005; Schwerd et al., 2017; Dike et al., 2019).

The clinical cases presented in this study are unique since the patients did not have any typical neurological symptoms at diagnosis but showed immune system hyperactivity in early infancy. In both patients, elevated immunoglobulins were noticed. In addition, there were multiple autoantibodies in the first patient. We excluded other possible factors that may cause immune activation. Before the immunoglobulin blood test, none of the patients were treated with any medications (*e.g.*, gamma globulin) that may have affected the immune system. The first patient’s mother was examined. There were no abnormalities in her immune function or symptoms of rheumatism, which ruled out the possibility of maternal antibody transmission through the placenta. However, we do not know whether the child may develop a secondary autoimmune disease, such as systemic lupus erythematosus, in the future.

NPC has no disease-specific treatment to modify the onset of neurologic progression or prolong lifespan. The disease usually progresses to premature death. Therefore, before the onset of neurological symptoms, treatment is essentially palliative and aims at alleviating specific symptoms (Geberhiwot et al., 2018; Matencio et al., 2020). The two infants included in this study mainly presented with cholestatic liver disease, which was treated with symptomatic support. In addition, given the presence of severe pneumonia and activation of immunity in the first patient, methylprednisolone, a common immunosuppressant drug, was used, and produced a good response. It indicates that methylprednisolone effectively treated the patient’s immune system irregularities. However, we do not know yet whether this treatment would be suitable for other similar cases. Indeed, the limitation of our study is that it is difficult to draw any wider conclusions regarding these findings.

In conclusion, this study reported two unique cases that presented the typical clinical features of infantile NPC and immune system hyperactivity. Genetic analysis of these patients further confirmed the diagnosis of NPC, and three novel NPC1 variants were identified by exome sequencing. Our findings suggest that recognizing immune activation in LSDs is essential, and expand both the clinical phenotype and genetic mutation spectrum of NPC1.

## Data Availability

The datasets for this article are not publicly available due to concerns regarding participant/patient anonymity. Requests to access the datasets should be directed to the corresponding authors.
